# Enhanced Solubility and Bioavailability of Apigenin via Preparation of Solid Dispersions of Mesoporous Silica Nanoparticles

**Published:** 2019

**Authors:** Yannian Huang, Xiuhua Zhao, Yuangang Zu, Lu Wang, Yiping Deng, Mingfang Wu, Huimei Wang

**Affiliations:** *Key Laboratory of Forest Plant Ecology, Northeast Forestry University, Ministry of Education Harbin, Heilongjiang 150040, China.*

**Keywords:** Apigenin, Mesoporous silica nanoparticles, Solid dispersion, Solubility, Dissolution, Bioavailability

## Abstract

In this study, a novel mesoporous silica nanoparticles drug carrier contributes to improving the solubility, dissolution, and the oral bioavailability of apigenin (AP). The apigenin of solid dispersion of mesoporous silica nanoparticles (AP-MSN) was prepared by physical absorption method and also, *in-vitro* drug release and *in-vivo* bioavailability performance were evaluated. Based on its solubility, the AP-MSN solid dispersion was prepared at the weight ratio of 1:1 to obtain the optimum solubility. The loading efficiency (LE), encapsulation efficiency (EE), and solubility of AP-MSN solid dispersion were 29.71%, 42.27%, and 25.11 µg/mL, respectively. SEM, TEM, BET, FTIR, XRD, DSC, and TG were also carried out. These results demonstrated that AP was good absorbed into the pores of MSN through physical absorption effect of MSN. The DMF residues of AP-MSN solid dispersion meet the ICH requirements. It was vital that the AP-MSN solid dispersion behaved well *in-vitro* and the accumulative release of AP-MSN solid dispersion was 2.37 times higher than that of raw AP. *In-vivo* study, the AP area under curve_0-t_ was 8.32 times higher for the AP-MSN solid dispersion than that of raw AP, which indicated that the bioavailability of AP-MSN solid dispersion was greatly improved. Therefore, the prepared AP-MSN solid dispersion presents potential as a novel oral therapeutic agent formulation for clinical application.

## Introduction

**Figure 1:**
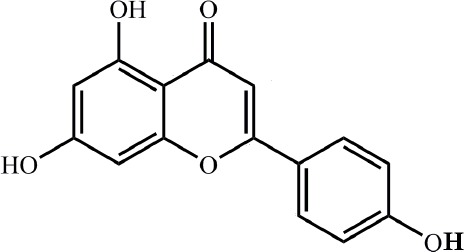
Chemical structure of apigenin

Apigenin[AP;4′,5,7-trihydroxyflavone; Figure 1], a common flavonoid, is widely distributed in most of vegetables and fruits, containing celery, chrysanthemum, orange and so on. AP attracts great attention because it has notable antioxidant, anti-cancer, and anti-inflammatory properties (1-6). AP has also the ability to modulate some expression processes such as PI3K-Akt, MAPKs, casein kinase-2, and other upstream kinases (7-10). However, AP, as BCS class II drugs, characterizes relatively low solubility and high permeability in intestine (11), which lead to extremely low bioavailability of AP (12, 13). Though AP behaves well in pharmacological aspect, the poor solubility of AP hinders its clinical application. Therefore, it is no time to delay developing new formulations or technologies to improve solubility of AP and then to better its bioavailability.

At the present, some researchers have formulated AP in some approaches to improve its solubility. The drug delivery system is as followed: liposomes (14, 15), AP-loaded self-microemulsifying drug delivery system (16) AP-loaded polymeric micelles (17), carbon nanopowder (18), nanosuspension (19) nanocrystal prepared by supercritical antisolvent process (20). In general, this drug delivery system has its own merits, which benefits improvement of the bioavailability of AP to some degree. Most of researchers have demonstrated that solid dispersion is widely used to enhance the solubility and dissolution of the poor water-soluble drugs and to solve other problems including instability and dosing (21-23). 

The selection of carriers is critical for properties of the final solid dispersion. Solid dispersion was classified into crystalline solid dispersion and amorphous solid dispersion depending on the physical state of the carriers. Moreover, the dispersions are also divided into four generations on the basis of their composition. The first generation is crystalline solid dispersion, which leads to low dissolution rate due to crystalline carrier and low stability. The second generation is amorphous solid dispersion containing amorphous solid solution (glass solutions) and amorphous solid suspensions based on the physical state of drugs. The third is amorphous solid dispersion with additives of the surface active agents or self-emulsifiers that can overcome the precipitation and recrystallization. 

The third solid dispersion contributes to the improvement of dissolution rate and the decrease of precipitation under supersaturation. The last generation solid dispersion is controlled release solid dispersion and these carriers are insoluble or low dissolved in water so that they can sustain the release of poor water-soluble drugs (21). Since the mesoporous silica nanoparticles are an insoluble material in water, so these carriers can be classified into the last generation solid dispersion. The mesoporous silica nanoparticles take possession of many advantages such as excellent biocompatibility and biodegradability, enormous surface area, the huge amount of pores, the pore size with narrow distribution, as well as good physicochemical stability (24). Therefore, mesoporous silica nanoparticles are widely applied to the field of biomedicine, catalyst supports, and biomaterial *etc.* (25). Mesoporous silica nanoparticles can control drug release and can be used as promising carrier for cancer therapy owing to their size tunability, surface functionality, optically transparent properties, low toxicity, and high drug loading efficiency (26-32). This drug nanocarrier will inspire develop of new smart and self-fluorescent drug carrier with imaging (32). In addition, incorporation of mesoporous silica nanoparticles into Poly (lactic-co-glycolic acid) and Poly (lactic-co-glycolic acid)/gelatin random nanofibrous scaffolds lead to improve cell attachment, proliferation, and enhance cellular processes (33). Even some researchers have demonstrated that mesoporous silica nanoparticles can enhance seedling growth and photosynthesis in wheat and lupin (34). Importantly, mesoporous silica has excellent properties to improve the oral bioavailability of poor water-soluble drugs (35). 

In this study, the weight ratio of mesoporous silica nanoparticles and raw AP was 1:0.5, 1:1, 1:2, 1:3 to investigate the effect of LE, EE, and solubility for AP-MSN solid dispersion. When optimum LE, EE, and solubility of AP-MSN solid dispersion had been obtained, scanning electron microscopy (SEM), transmission electron microscopy (TEM), Brunauer, Emmett and Teller (BET), fourier transform infrared spectroscopy (FTIR), X-ray diffraction (XRD), differential scanning calorimetry (DSC) and thermal gravimetric (TG), and gas chromatograph (GC) measurement were analyzed later. Then, the dissolution and oral bioavailability of AP-MSN solid dispersion were also carried out. 

## Experimental

*Material*

Apigenin (purity 98%) was purchased from Baoji Haoxiang Bio-technology Co., Ltd. (Shanxi, 99 China). Mesoporous silica nanoparticles (purity 99.8%), with the particle size of 7-40 nm, was provided by Aladdin Industrial Corporation (Shanghai, China). Methanol (chromatography grade) was purchased from Chang Tech Enterprise Co., Ltd in China. The acetone (chromatography grade) and sodium dodecyl sulfate were all purchased from Sinopharm Chemical Reagent Co., Ltd in China. Ethanol and N, N-dimethylformamide (DMF purity > 99.5%) were offered by Tianjin Zhiyuan Chemical Reagent Co., Ltd and Tianjin Fuyu Fine Chemical Co., Ltd, respectively. 

*Preparation of AP-MSN nanoparticles solid dispersion*

*Activation of MSN*

In this study, mesoporous silica nanoparticles (MSN) are selected as solid dispersion carrier to prepare AP-MSN solid dispersion. MSN are a neotype inert nano material. It has many pores and does not generate reaction with active substance. Before experiment, the MSN has to be activated to expel air of pores, which contributed to improving the drug loading. The requirements of activation are that the MSN in vacuum environment was maintained for 6 h at 1 Pa and 25 °C. 

*Preparation of AP-MSN solid dispersion*

AP-MSN solid dispersion was prepared by physical absorption method. AP were dissolved in 15 mL of DMF with a concentration of 120 mg/mL and then MSN dispersed in AP of 120 mg/mL solution with AP and MSN at the weight ratio of 1:0.5, 1:1, 1:2, 1:3. Then the mixtures of AP and MSN were sufficiently stirred at room temperature for 1 h. After stirring, 250 mL deionized water was respectively poured into the mixtures of AP and MSN, and then all of these mixtures were filtered by 0.22 µm organic filter membrane to abandon filtrate and get the residues, respectively. These residues were respectively dispersed in 250 mL ethanol and then filtered by 0.22 µm organic filter membrane again to attain the ultimate residues (AP-MSN solid dispersion). The ultimate residues (AP-MSN solid dispersion) were dried at 40 °C for 12 h. The first and second each filtrate containing fraction AP were assayed using high performance liquid chromatography (HPLC) method to get loading efficiency (LE) and encapsulation efficiency (EE) of AP-MSN solid dispersion. The LE of AP-MSN solid dispersion was calculated as the ratio between the actual AP content and the AP-MSN solid dispersion, and it was expressed as a percentage. The EE of AP-MSN solid dispersion was the ratio of actual AP content and theoretical amount of AP in AP-MSN solid dispersion, which was also expressed by percentage. The physical mixture (PM) of AP and MSN were prepared by mixing AP with MSN at the weight ratio of 1:1 and then thoroughly grinded by a mortar and pestles.

$\mathrm{LE}\left(\%\right)=\frac{\mathrm{mass of actual AP}}{\mathrm{mass of AP}-\mathrm{MSN solid dispresion}}\times 100$

$\mathrm{EE}\left(\%\right)=\frac{\mathrm{mass of actual absorbed AP}}{\mathrm{mass of total AP}}\times 100\%$

*High performance liquid chromatography (HPLC) analysis of AP*

HPLC (Waters Corporation, Milford, MA, USA) was used to determine AP content about these aspects such as LE, solubility, dissolubility, and bioavailability of AP. The chromatographic column was a Diamonsil C_18_ ­_­­­_reverse-phase column (250 mm × 4.6 mm, 5 μm, China) at indoor temperature. All samples were centrifuged at 10000 rpm/min for 10 min. Samples of 10 μL volume were injected into the C_18_ reverse-phase column using a mobile phase consisting of a 70:30:0.2% mixtures of methanol, water, and phosphoric acid. The detection wavelength was 353 nm (the type and model of detector: Waters 2489, UV/Visible Detector). The linear calibration curve was obtained in the concentration range from 50 to 0.098 µg/mL, with a correlation coefficient of 0.9997.

*Scanning electron microscopy (SEM), particle size and transmission electron microscopy (TEM) analysis*

The morphological characteristic of raw AP and AP-MSN solid dispersion (weight ratio, 1:1) were observed by SEM apparatus (Quanta 200, FEI, The Netherlands). Before measurement, the samples were fixed on SEM stubs through double-sided conducting adhesive tape coated with a thin layer of gold. The morphology of samples was analyzed. 

The TEM measurements were performed using the JEM-100CXII Transmission Electron Microscope (Hitachi, Ltd., The Japan). First, the amount of the AP-MSN solid dispersion was equably dispersed in deionized water. Then, few droplets of the suspension were placed on the copper grid, which followed by drying under ambient conditions in an Ar glovebox. The copper grid containing samples were transferred to the microscope using a special vacuum-transfer sample holder under exclusion of air. The shape of sample was analyzed finally.

The particle size were determined by Dynamic light scattering (DLS) (ZetaPALS, Brookhaven instruments) with a He-Ne laser (632.8 nm, 35 mW) as light source. The sample was prepared by dilution of AP-MSN solid dispersion in pure water and then was sonicated 30 sec. 

*Brunauer, Emmett and Teller (BET) surface area analysis*

The BET surface area of samples was determined using nitrogen gas adsorption at ASAP2020 Automated adsorption apparatus (Micrometric Ltd, USA) after degassing the samples in a flowing nitrogen atmosphere at 300 °C for 10 h. According to the relative pressure range of 0.01-1.0 using the Brunauer, Emmett and Teller (BET), the specific surface area was obtained. 

*Fourier transform infrared spectroscopy (FTIR) analysis*

FTIR spectrometer (Shimadzu Corporation, Japan) was used to analyze and identify the molecular structure of the samples. The test procedures were as followed: the samples and KBr were dried at 105 °C for 1 h, and then the mixture samples, mixed with KBr at the weight ratio of 1:99 (the total weight, 200 mg) were pressed into pellucid flakes. Finally, the pellucid flakes were detected in wave numbers at 400−4000 cm^-1^ at a resolution of 4 cm^-1^. 

*X-ray diffraction (XRD) analysis*

The samples were analyzed by XRD pattern using an X-ray powder diffractometer with a rotating anode (Philips, Xpert-Pro, Netherlands) and Cu Ka1 radiation generated at 30 mA and 50 kV. The scanning rate was 5 °/min from 5 to 60 °.

*Differential scanning calorimetry (DSC) analysis*

DSC measurements were performed using DSC (TA instruments, model DSC 204, New Castle in USA) to get DSC curves of AP, MSN, AP-MSN solid dispersion and PM of AP and MSN. All thermal analysis were conducted in N_2_ atmosphere at a heating rate of 10 °C min^-1^ ranging from 30 °C to 300 °C. 

*Thermal gravimetric (TG) analysis*

The thermal stabilities of samples were analyzed with a thermogravimetric analyzer (TGA, Diamond TG/DTA PerkinElmer, USA). Briefly, 5 mg of the samples were heated at a rate of 10 °C min^-1^ from 30 °C to 400 °C under dynamic nitrogen atmosphere. 

*Gas chromatography (GC) measurement*

The residual DMF and enthanol in AP-MSN solid dispersion were detected using a gas chromatograph (GC) (Agilent 7890A, Palo Alto, CA, USA), equipped with a G1540-210 flame ionization detector and a HP-5 5% phenyl methyl siloxane capillary column (30.0 m × 320 mm × 0.25 µm). Twenty mg of the AP-MSN solid dispersion was dispersed in 1 mL of acetone, which was ultrasonically treated for 15 min. Then, the suspension was centrifuged for 10 min at 8944 g to attain the supernatant. Two μL of the supernatant was injected into the GC column and the peak area of the curve was used for getting quantitative data. The detection conditions for GC analysis of DMF were as follows: initial oven temperature of 40 °C for 5 min, increasing to 240 °C at a rate of 40 °C/min, and maintaining the temperature for 15 min; injector temperature of 200 °C; detector temperature of 280 °C; the H_2_ rate was set to 30 mL/min; the airflow rate was set to 400 mL/min; and N_2_ was used as carrier gas at a rate of 2.2 mL/min. The sample (2 µL) was directly injected in split mode at a split ratio of 20:1.

*Solubility of AP in AP-MSN solid dispersion*

The solubility study was carried out and assayed using the HPLC method. Excess raw AP and each batch AP-MSN solid dispersion (AP and MSN weight ratios, 1:0.5, 1:1, 1:2, 1:3) were placed into 15 mL beakers containing 5 mL of 0.4% sodium dodecyl sulfate, respectively. Then, the beakers were sealed by sealing film, and the suspensions were stirred by magnetic stirring apparatus for 48 h under 100 ± 2 rpm at 37 °C. After stir, the suspensions were filtered by 0.22 µm membranes, respectively. The filtrates were respectively diluted with 9 mL of methanol, and then were ultrasonically treated for 10 min before assayed by HPLC method.

*In-vitro* *dissolution study*

*In-vitro* dissolution test was performed. Weighing out 6.78 mg of AP-MSN solid dispersion (equivalent to 2 mg of AP) and 2 mg of raw AP were respectively added to 2 mL of 0.4% sodium dodecyl sulfate dissolution medium to obtain the suspensions. The raw AP suspension and AP-MSN solid dispersion suspension were respectively placed into the treated dialysis bags (MWCO 3500; Sigma, St. Louis, USA), and then the both ends of dialysis bags were tightly clamped. The dialysis bags were respectively immersed in two identical 500 mL beakers containing 400 mL 0.4% sodium dodecyl sulfate as dissolution medium. The two beakers were shaken at 100 ± 2 rpm and 37 °C by using a magnetic stirrer. The dissolution medium (5 mL) was obtained at predetermined time interval (15 min, 30 min, 45 min, 60 min, 90 min, 120 min, 150 min, 3 h, 4 h, 6 h, 8 h, 12 h, 1 day, 2 day), and 5 mL of fresh dissolution medium was placed into the beaker to maintain a constant volume. One mL of dissolution medium solution was taken out and mixed with 4 mL of methanol subsequently. The mixture was ultrasonically treated for 10 min, and then centrifuged at 10000 rpm/min for 10 min. Finally, the 10 µL of supernatant was injected into HPLC apparatus. The detailed analysis conditions were described in section 2.3.

**Table 1:** The LE and EE of AP-MSN solid dispersion

**AP/MSN weight ratio**	**LE (%)**	**EE (%)**
1:0.5	44.69	40.4
1:1	29.71	42.27
1:2	17.87	43.51
1:3	13.14	45.39

**Table 2.** BET surface area

**Factor**	**sample**	**surface area (m** **2** **/g)**
1	MSN	235.74
2	AP-MSN solid dispersion	169.41

**Figure 2:**
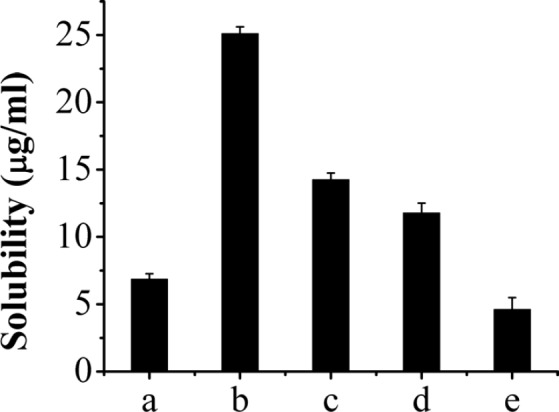
The solubility of AP-MSN solid dispersion and raw AP. AP-MSN solid dispersion at AP/MSN weight ratio of (a) 1:0.5, (b) 1:1, (c) 1:2, (d) 1:3, and (e) raw AP

**Figure 3:**
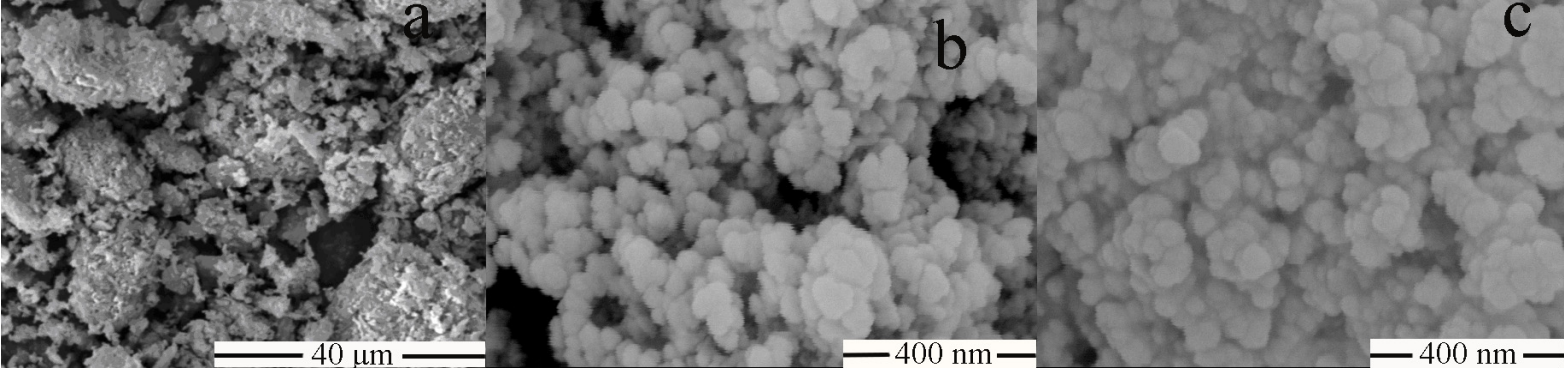
SEM images of (a) raw AP, (b) MSN and (c) AP-MSN solid dispersion

**Figure 4:**
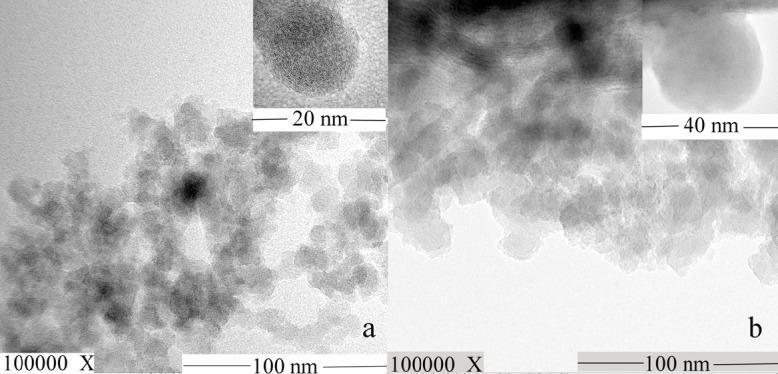
TEM images of (a) MSN and (b) AP-MSN solid dispersion

**Figure 5:**
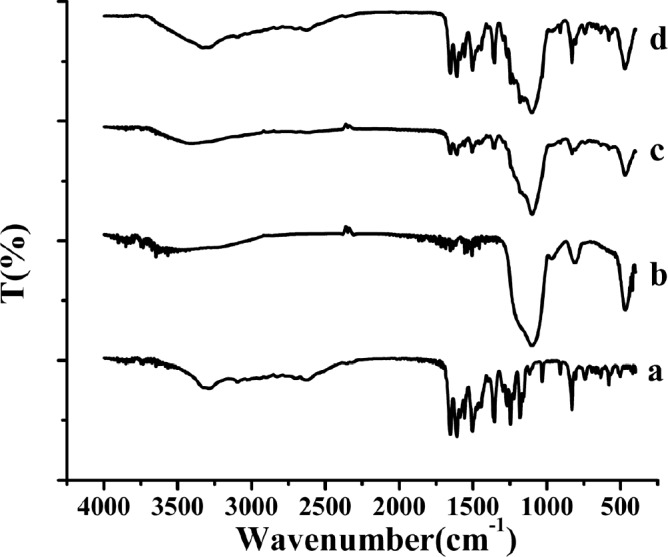
FTIR spectra of the samples. (a) raw AP, (b) MSN, (c) AP-MSN solid dispersion, (d) physical mixture of AP/MSN 1:1

**Figure 6:**
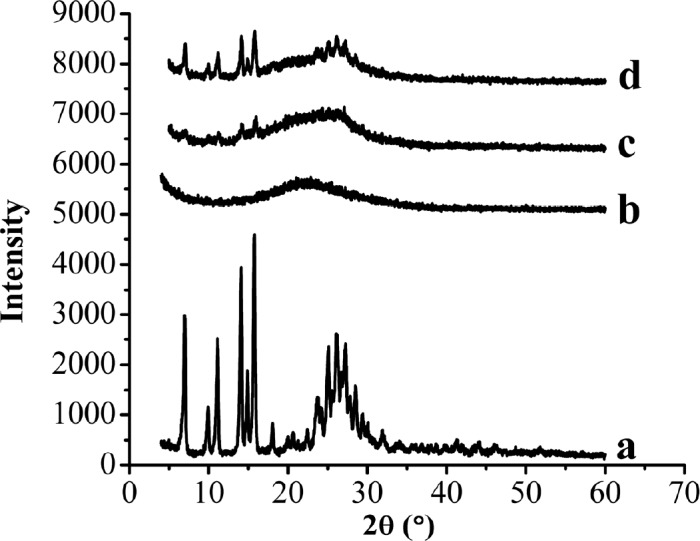
XRD patterns of the samples. (a) Raw AP, (b) MSN, (c) AP-MSN solid dispersion, (d) physical mixture of AP/MSN 1:1

**Figure 7:**
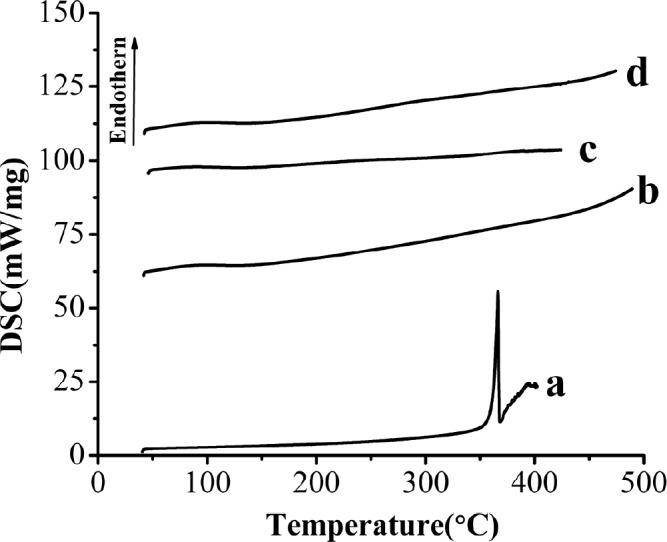
DSC curves of the samples. (a) Raw AP, (b) MSN, (c) AP-MSN solid dispersion, (d) physical mixture of AP/MSN 1:1

**Figure 8:**
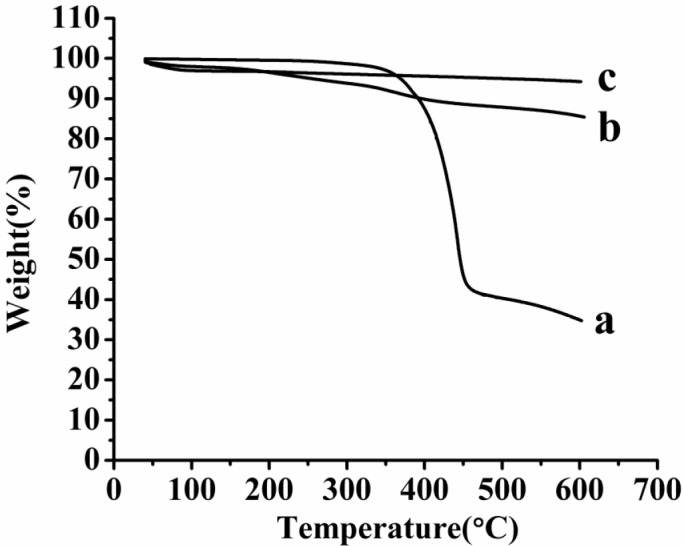
TG thermogram of the samples. (a) AP, (b) AP-MSN solid dispersion, (c) MSN

**Figure 9:**
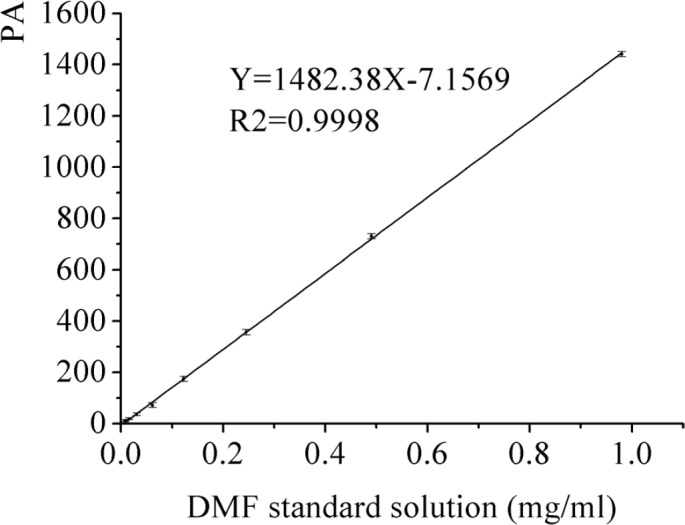
The calibration curve of DMF standard solution

**Figure 10:**
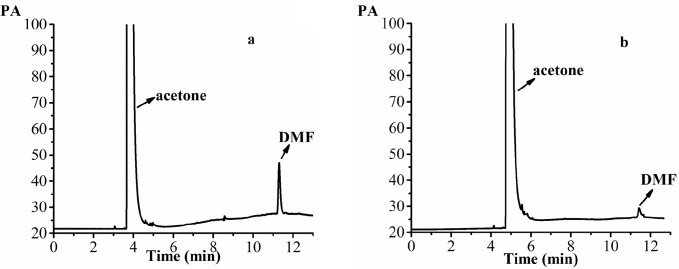
The GC patterns of the samples. (a) 0.0375 mg/mL DMF standard solution (acetone as solvent), (b) 20 mg/mL acetone solution of AP-MSN solid dispersion (acetone as solvent)

**Figure 11:**
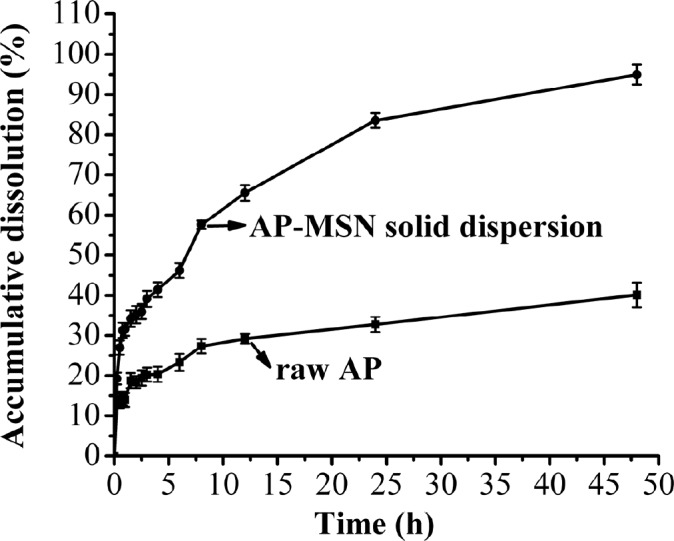
Dissolution of AP-MSN solid dispersion and raw AP

**Figure 12:**
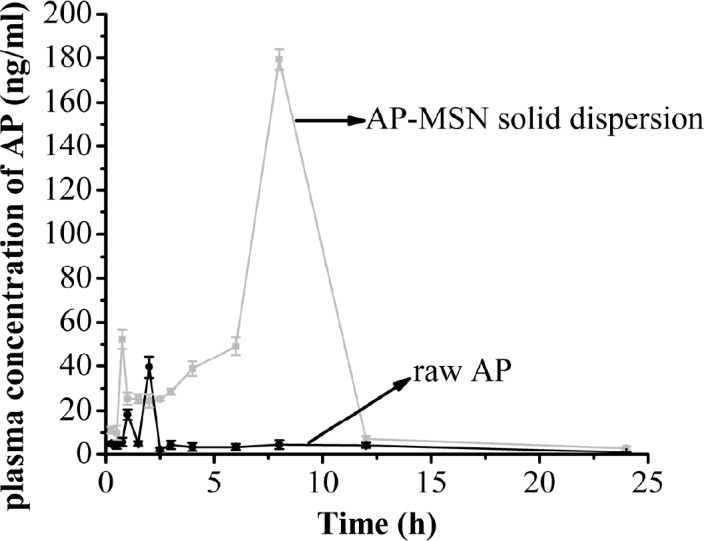
The plasma concentration of AP-MSN solid dispersion and raw AP

*Bioavailability*

*Animals experiments*

The all animal experiments were reviewed and approved by the Ethical Committee of Northeast Forestry University. Female Sprague-Dawley rats (weight, 220 ± 10 g) were obtained from Experimental Animal Center of the Harbin (Harbin, China). On account of variation of environment, the rats had to spend least a week on adapting to experimental environment that the humidity and temperature were 55% ± 5% and 25 ± 2 °C, respectively. Twelve female Sprague-Dawley rats, weighting range of 210-230 g, were randomly divided into two groups with six rats in each one. Before experiment, the rats were subjected to a night of nothing food with sufficient water. 

The oral formulation of raw AP and AP-MSN solid dispersion for rats was at the dose equivalent of 60 mg/kg AP. The given amount of raw AP and AP-MSN solid dispersion were respectively dispersed into 3 mL of deionized water and then the suspension was administrated by gavage. The blood samples were collected by puncturing the orbital venous sinus and placed into tubes that contained 1% heparin. The blood samples in tubes should be shaken slightly and then centrifuged at 3000 rpm/min for 10 min. The supernatant serum samples were removed from the tubes into new tubes and stored at −40 °C until analysis. 

*Analysis of AP in rat plasma*

The frozen serum samples could be thawed at room temperature. The 200 µL of serum sample mixed with 600 µL of methanol and then the mixture was in vortexes for 3 min to extract AP. The mixture was centrifuged at 10000 rpm/min for 10 min and then the supernatant was transferred to another tube and set aside. Another 600 µL of methanol was added to the residues, vortex-mixed for 3 min, and centrifuged at 10000 rpm/min for 10 min again. The second supernatant was put together with the first supernatant. 

The obtained supernatant was dried by a slight stream of nitrogen to attain the residues. The residues was dissolved in 100 µL of methanol and centrifuged at 10000 rpm/min for 10 min before analysis in HPLC system. The HPLC method was described in the section 2.3.

## Results and Discussion

*AP content and solubility study*

The LE and EE of AP-MSN solid dispersion were shown in Table 1. The LE of AP-MSN solid dispersion was decreased with the increase of MSN. However, the EE of AP-MSN solid dispersion presented the trend of ascent with increasing MSN. MSN had many pores and electronegativity, therefore, they had great advantages of absorbing other substances. The amount of MSN was bigger, the more AP was absorbed. So the EE of AP-MSN solid dispersion increased along with an increase of MSN. But it was difficult to choose the best AP-MSN solid dispersion; the solubility should be tested for all solid dispersions. 

The AP-MSN solid dispersions (AP and MSN weight ratio, 1:0.5, 1:1, 1:2, 1:3) solubility were also presented in Figure 2. It was obvious that the best solubility was 25.11 µg/mL belonging to the AP-MSN solid dispersion at the weight ratio of 1:1. This solubility of AP-MSN solid dispersions was greater than that of raw AP because of the disorder structure of amorphous AP-MSN solid dispersion (36). The study about apigenin-loaded polymeric micelles was carried out (17) and demonstrated that the solubility of aigenin-loaded polymeric micelles was 2.16 µg/mL and its LE merely 1.32%. Compared to the previous study, the solubility and LE of AP-MSN solid dispersion were higher. Therefore, the solid dispersion with an AP/MSN weight ratio of 1:1 was selected for the further study. 

*Characterization of AP-MSN solid dispersion*

*Morphology study*

Figure 3 showed SEM images of raw AP, MSN, and AP-MSN solid dispersion. The raw AP was shown in Figure 3a, which was a shape of irregular bulk with the range diameters between 5 μm and 20 μm. The mesoporous silica nanoparticles (Figure 3b), present in form of spherical, had a regular shape. From Figure 3c, the shape of AP-MSN solid dispersion was in accordance with that of mesoporous silica nanoparticles. 

To further testify that the AP dispersed in the pore of MSN, the TEM images (Figure 4) of MSN and AP-MSN solid dispersion were attained by observation with TEM apparatus. Figure 4a showed that MSN with a uniform sphere and smooth surface had extremely small diameter (smaller than 20 nm). AP-MSN solid dispersion (Figure 4b) was in the form of spherical shape and similar to the shape of MSN mostly. Since the small amount of AP existed on surface of MSN, the size of AP-MSN solid dispersion was added to 30 nm approximately. The AP-MSN solid dispersion was determined via DLS method. The AP-MSN solid dispersion particle size, zeta potential, and polymer dispersity index were 49 nm, -15.0 mV, and 0.113, respectively. These results indicated that AP-MSN solid dispersion was formed.

Table 2 showed BET surface of MSN and AP-MSN solid dispersion. The surface area of MSN and AP-MSN solid dispersion were 235.74 and 169.41 m^2^/g, respectively. It was apparent that the surface area of MSN became smaller after absorption of AP. These results revealed that the AP was mostly dispersed in the pores of MSN resulting in the smaller area surface of AP-MSN solid dispersion. Surely, the surface area of MSN was the largest. However, the surface area of AP-MSN solid dispersion was smaller than that of MSN, which reflected the LE of AP-MSN solid dispersion.

*FTIR analysis*

The FTIR spectra of raw AP, MSN, AP-MSN solid dispersion, and physical mixture of AP/MSN were shown in Figure 5. The spectra of raw AP in Figure 5a showed that there was a broad band at 3290 cm^-1^ originating from the valence vibration of (O-H) groups in the structure of raw AP, which were likely to be related with the formation of intramolecular hydrogen bands with the C=O group of the ring. Some obvious intensive bands at 1652, 1608, and 1504 cm^-1 ^could prove the existence of C=O groups in the structure of raw AP. 

The other intensive band at 1354, 1246, 1182, 1031, and 908 cm^-1^ was generated as the result of the C-O groups and deformation (C-OH) variation from the structure of raw AP. In Figure 5b, there were three main intensive bands at 1093 cm^-1^ (Si-O- Si anti-symmetrical stretching vibration) (37), 806.2 cm^−1^ and 466.7 cm^−1^ (Si-O flexural vibrations and symmetrical stretching vibration) (38). By comparison of Figures 5a, 5b, and 5c, the peaks (Figure 5c) were little obvious at 1608, 1504, 1354, and 829 cm^−1^ originating from AP in the AP-MSN solid dispersion and some peaks (Figure 5a) at 1031, 908, 742, 578, and 501 cm^−1^ from AP were almost in the absence of AP-MSN solid dispersion. Comparing MSN with AP-MSN solid dispersion, the peaks originating from MSN were hardly changing except for the peak at 806.2 cm^−1^. Therefore, the AP was well dispersed inside the pores of MSN. In contrast with curves of Figures 5a, 5b, and 5d, the peaks (Figure 5d), originating from AP in PM of AP/MSN, had distinct peaks in step with the peaks of raw AP (Figure 5a), indicating that AP was merely attached to the surface of MSN. All above results indicated that AP-MSN solid dispersion was formed to some degree.

*XRD analysis*

The XRD patterns of raw AP, MSN, AP-MSN solid dispersion, and PM of AP/MSN (1:1) were shown in Figure 6. Figures 6a and 6b showed that the raw AP exhibited some characteristic peaks at angles of diffraction, whereas no distinct peaks were detected in the XRD pattern of the MSN. Compared with the XRD patterns of raw AP and MSN in Figures 6a and 6b, respectively, the XRD pattern of AP-MSN solid dispersion, as shown in the Figure 6c, was nearly absent in any diffraction peak in accordance with the raw AP. However, the XRD pattern of PM of AP/MSN (1:1) had some diffraction peaks that were not only similar to the diffraction peaks of raw AP but also identical to the diffraction peaks of the MSN. 

These results suggest that AP-MSN solid dispersion was not present in the form of crystalline, but rather existed in an amorphous state. Therefore, through examination of XRD, AP-MSN solid dispersion was attained and the AP was almost amorphous state in AP-MSN solid dispersion. These outcomes further proved the results of DSC analysis.

*DSC analysis*

Figure 7 showed the results of the DSC analysis of raw AP, MSN, AP-MSN solid dispersion and PM of AP/MSN (1:1). The DSC pattern of raw AP (Figure 7a) had a distinct endothermic peak at about 366.36 °C in line with the melting point of AP, which demonstrated crystalline structure of AP in nature. This was corresponded to the result reported by Zhang *et al.* (20). However, the curve of MSN in Figure 7b had no peak, so it indicated that MSN had been in an amorphous form. In contrast with Figures 7a and 7b, the curve of AP-MSN solid dispersion in Figure 7c had no obvious peak at all, which was basically the same with the curve of MSN. 

This result implied that AP was molecularly dispersed into the pores of MSN and might have existed in the form of an amorphous state, which facilitated solubility and dissolution. Figure 7d showed that the curve of PM of AP/MSN (1:1) was similar to the curve of AP-MSN solid dispersion. It was possible that AP was absorbed into the pores of MSN on account of high temperature for PM of AP/MSN (1:1) by DSC process, but their differences were analyzed by XRD, as shown in section of XRD analysis. 

*TG analysis*

Figure 8 showed the TG thermogram of raw AP, MSN and AP-MSN solid dispersion. When the temperature was 600 °C, the weight remain of raw AP, MSN and AP-MSN solid dispersion were 34.93, 94.25, and 85.64%, respectively. On account of characteristic of MSN, When AP was absorbed in pore of MSN, the weight remain of MSN and AP-MSN solid dispersion had the less difference at the temperature of 600 °C compared with the difference about the weight remaining between AP and AP-MSN solid dispersion. Account for LE of AP-MSN solid dispersion, the weight detainment of MSN and AP-MSN solid dispersion was different. Comparing with Figures 8a, 8b and 8c, the Figure 8a had a distinct inflection point at 366.36 °C; however, Figures 8b and 8c had no inflexion at all. These results implied that the AP was almost amorphous in AP-MSN solid dispersion.

*Solvent residue*

DMF is classified as a class Ⅱ solvent and the quantity of DMF in dug is less than 0.088% by the International Conference on Harmonization (ICH). The DMF as solvent was used for the preparation of AP-MSN solid dispersion. The DMF residues in AP-MSN solid dispersion was detected by GC and its residues was 0.055% by calculation according to the regression equation (Y = 1482.38X - 7.1569, where X is DMF concentration and Y is the peak area, in Figure 9). Figure 10 showed that DMF residues had sharp peak at 11.30 min by determination of GC. The DMF residues in AP-MSN solid dispersion meet the ICH requirements and may be suitable for the pharmaceutical use.

*In-vitro release study*

*In-vitro* dissolution profiles of the AP-MSN solid dispersion and raw AP are shown in Figure 11. The dissolution of AP-MSN solid dispersion behaved better than that of raw AP. Not only the higher the dissolution velocity of the AP-MSN solid dispersion than that of raw AP, but also the AP-MSN solid dispersion also achieved higher accumulation of dissolved AP. Specially, the AP-MSN solid dispersion had great effect on control release and its biggest cumulative dissolution got 95.2% at 48 h. The effects of control release about AP-MSN solid dispersion are attributed to MSN with many pores being useful for absorption of drug. Moreover, AP might be formed in amorphous state in AP-MSN solid dispersion, which contributed to improve cumulative dissolution of AP. However, the raw AP had low cumulated dissolution at 48 h, with mere 40.11%. In general, the AP-MSNA solid dispersion stilled showed a good performance for dissolution.

*Bioavailability study*

The bioavailability results of raw AP and AP-MSN solid dispersion are shown in Figure 12. From 0 to 0.75 h, the concentration of AP in the rat plasma of AP-MSN solid dispersion ascended excepting for slight decline at the range of 0.25-0.5 h, and it achieved bigger value (52.17 ng/mL) at 0.75 h. However, the concentration of AP in rat plasma of AP-MSN solid dispersion decreased all the way from 0.75 to 1 h and then had little variation from 1 to 2.5 h. On account of effect about MSN, the concentration of AP in rat plasma increased from 2.5 to 8 h and achieved maximum (179.39 ng/mL) at 8 h. On the other hand, from 8 to 24 h, the concentration of AP in rat plasma of AP-MSN solid dispersion continuously declined. As for the raw AP, the concentration of raw AP in rat plasma was low with the exception of concentration of 18.21 ng/mL and 39.59 ng/mL at 1h and 2 h, respectively. The oral relative bioavailability of AP was calculated and represented by AUC (area under the curve) values. The AUCs of AP-MSN solid dispersion and raw AP were 854.89 and 102.69 µg/Lh, respectively. These results demonstrated that the oral relative bioavailability of AP-MSN solid dispersion increased by 8.32 times by comparison of that of the raw AP. The AUC of AP nanocrystal prepared by SAS method (20) was 3.4 times, which was higher than that of the raw AP. Furthermore, by comparison of raw AP, solid dispersion AP with carbon nanopowder (18) had an AUC 1.83 times higher. Comparing to previous study in bioavailability, the AP-MSN solid dispersion took better advantage over raw AP. Therefore, the AP-MSN solid dispersion showed an improvement on the oral relative bioavailability of AP.

AP nanocrystals prepared by SAS method (20) had a good effect on bioavailability, with AUC 3.4 times higher than that of raw AP. In addition, compared to raw AP, solid dispersion of AP with carbon nanopowder had an AUC 1.83 times higher (18). However, in comparison to these previous studies in bioavailability, the present study on the AP-MSN solid dispersion showed a more significant effect compared with that of the raw AP.

## Conclusion

In this study, the weight ratio of AP and MSN (1:1) was established by physical absorption method based on LE, EE, and solubility for AP. The LE and EE of AP-MSN solid dispersion were respectively 29.71% and 42.27% and its solubility was 25.11 µg/mL higher than that of raw AP. Then, the characteristics of AP-MSN solid dispersion were also analyzed by SEM, TEM, BET, FTIR, XRD, DSC, and TG, and their results imply that the AP was almost absorbed in the pores of MSN and that the AP-MSN solid dispersion was formed absolutely. DMF residues in the AP-MSN solid dispersion was 0.055% lower than the ICH limit for the class II solvents. The dissolution study demonstrated that AP-MSN solid dispersion had a good dissolution velocity compared with raw AP. Furthermore, the bioavailability study showed that the oral relative bioavailability of AP-MSN solid dispersion was 8.32 times as many as that of raw AP. These results demonstrated that the formation of AP-MSN solid dispersion was successful to improve the dissolution performance and the oral relative bioavailability of AP. Therefore, AP-MSN solid dispersion has good prospect to be used as novel oral formulation for clinical application. 

## References

[1] Skerget M, Kotnik P, Hadolin M, Hras HR, Simonic M, Knez Z (2005). Phenols, proanthocyanidins, flavones and flavonols in some plant materials and their antioxidant activities. Food Chem.

[2] Tong X, Van Dross RT, Abu-Yousif A, Morrison AR, Pelling JC (2007). Apigenin prevents UVB-induced cyclooxygenase 2 expression: coupled mRNA stabilization and translational inhibition. Mol. Cell. Biol.

[3] Patel D, Shukla S, Gupta S (2007). Apigenin and cancer chemoprevention: Progress, potential and promise (Review). Int. J. Oncol.

[4] Zhu Y, Mao Y, Chen H, Lin Y, Hu Z, Wu J, Xu X, Qin J, Xie L (2013). Apigenin promotes apoptosis, inhibits invasion and induces cell cycle arrest of T24 human bladder cancer cells. Cancer Cell Int.

[5] Zhu Y, Wu J, Li S, Wang X, Liang Z, Xu X, Hu Z, Lin Y, Chen H, Qin J, Mao Q, Xie L (2015). Apigenin inhibits migration and invasion via modulation of epithelial mesenchymal transition in prostate cancer. Mol. Med. Rep.

[6] Patil RH, Babu RL, Naveen Kumar M, Kiran Kumar KM, Hegde SM, Nagesh R, Ramesh GT, Sharma SC (2016). Anti-Inflammatory effect of apigenin on LPS-Induced pro-inflammatory mediators and AP-1 factors in human lung epithelial cells. Inflammation.

[7] Fang J, Xia C, Cao ZX, Zheng JZ, Reed E, Jiang BH (2005). Apigenin inhibits VEGF and HIF-1 expression via PI3K/AKT/p70S6K1 and HDM2/p53 pathways. FASEB J.

[8] Gopalakrishnan A, Xu CJ, Nair SS, Chen C, Hebbar V, Kong AN (2006). Modulation of activator protein-1 (AP-1) and MAPK pathway by flavonoids in human prostate cancer PC3 cells. Arch. Pharm. Res.

[9] Hessenauer A, Montenarh M, Gotz C (2003). Inhibition of CK2 activity provokes different responses in hormone-sensitive and hormone-refractory prostate cancer cells. Int. J. Oncol.

[10] Lee WJ, Chen WK, Wang CJ, Lin WL, Tseng TH (2008). Apigenin inhibits HGF-promoted invasive growth and metastasis involving blocking PI3K/Akt pathway and beta 4 integrin function in MDA-MB-231 breast cancer cells. Toxicol. Appl. Pharm.

[11] Zhang J, Liu D, Huang Y, Gao Y, Qian S (2012). Biopharmaceutics classification and intestinal absorption study of apigenin. Int. J. Pharm.

[12] Xiao M, Shao YD, Yan WD, Zhang ZZ (2011). Measurement and correlation of solubilities of apigenin and apigenin 7-O-rhamnosylglucoside in seven solvents at different temperatures. J. Chem. Thermodyn.

[13] Xiao M, Yan WD, Zhang ZZ (2010). Solubilities of apigenin in ethanol plus water at different temperatures. J. Chem. Eng. Data.

[14] Banerjee K, Banerjee S, Das S, Mandal M (2015). Probing the potential of apigenin liposomes in enhancing bacterial membrane perturbation and integrity loss. J. Colloid Interface Sci.

[15] Ding BY, Chen H, Wang C, Zhai YJ, Zhai GX (2013). Preparation and in-vitro evaluation of apigenin loaded lipid nanocapsules. J. Nanosci. Nanotechnol.

[16] Zhao LL, Zhang L, Meng L, Wang J, Zhai GX (2013). Design and evaluation of a self-microemulsifying drug delivery system for apigenin. Drug Dev. Ind. Pharm.

[17] Zhai YJ, Guo SS, Liu CH, Yang CF, Dou JF, Li LB, Zhai GX (2013). Preparation and in-vitro evaluation of apigenin-loaded polymeric micelles. Colloid Surf. A.

[18] Ding SM, Zhang ZH, Song J, Cheng XD, Jiang J, Jia XB (2014). Enhanced bioavailability of apigenin via preparation of a carbon nanopowder solid dispersion. Int. J. Nanomedicine.

[19] Al Shaal L, Shegokar R, Muller RH (2011). Production and characterization of antioxidant apigenin nanocrystals as a novel UV skin protective formulation. Int. J. Pharm.

[20] Zhang JJ, Huang YT, Liu DP, Gao Y, Qian S (2013). Preparation of apigenin nanocrystals using supercritical antisolvent process for dissolution and bioavailability enhancement. Eur. J. Pharm. Sci.

[21] Vo CL, Park C, Lee BJ (2013). Current trends and future perspectives of solid dispersions containing poorly water-soluble drugs. Eur. J. Pharm. Biopharm.

[22] Kim MS, Kim JS, Park HJ, Cho WK, Cha KH, Hwang SJ (2011). Enhanced bioavailability of sirolimus via preparation of solid dispersion nanoparticles using a supercritical antisolvent process. Int. J. Nanomedicine.

[23] Kim SA, Kim SW, Choi HK, Han HK (2013). Enhanced systemic exposure of saquinavir via the concomitant use of curcumin-loaded solid dispersion in rats. Eur. J. Pharm. Sci.

[24] Hoffmann F, Cornelius M, Morell J, Froba M (2006). Silica-based mesoporous organic-inorganic hybrid materials. Angew. Chem. Int. Edit.

[25] Anglin EJ, Cheng LY, Freeman WR, Sailor MJ (2008). Porous silicon in drug delivery devices and materials. Adv. Drug Deliv. Rev.

[26] Jiao J, Li X, Zhang S, Liu J, Di DH, Zhang Y, Zhao QF, Wang SL (2016). Redox and pH dual-responsive PEG and chitosan-conjugated hollow mesoporous silica for controlled drug release. Mat. Sci. Eng. C-Mater.

[27] Feng Y, Panwar N, Tng DJH, Tjin SC, Wang K, Yong KT (2016). The application of mesoporous silica nanoparticle family in cancer theranostics. Coord. Chem. Rev.

[28] Wu L, Zhang HJ, Wu MH, Zhong YF, Liu XW, Jiao Z (2016). Dual-templating synthesis of multi-shelled mesoporous silica nanoparticles as catalyst and drug carrier. Micropor. Mesopor. Mat.

[29] Bouchoucha M, Cote MF, C-Gaudreault R, Fortin MA, Kleitz F (2016). Size-controlled functionalized mesoporous silica nanoparticles for tunable drug release and enhanced anti-tumoral activity. Chem. Mater.

[30] Paris JL, de la Torre P, Manzano M, Cabanas MV, Flores AI, Vallet-Regi M (2016). Decidua-derived mesenchymal stem cells as carriers of mesoporous silica nanoparticles In-vitro and in-vivo evaluation on mammary tumors. Acta Biomater.

[31] Ge K, Ren HH, Sun WT, Zhao Q, Jia G, Zang AM, Zhang CM, Zhang JC (2016). Walnut kernel-like mesoporous silica nanoparticles as effective drug carrier for cancer therapy in-vitro. J. Nanopart. Res.

[32] Xu XB, Lu SY, Gao CM, Feng C, Wu C, Bai X, Gao NN, Wang ZY, Liu MZ (2016). Self-fluorescent and stimuli-responsive mesoporous silica nanoparticles using a double-role curcumin gatekeeper for drug delivery. Chem. Eng. J.

[33] Mehrasa M, Asadollahi MA, Nasri-Nasrabadi B, Ghaedi K, Salehi H, Dolatshahi-Pirouz A, Arpanaei A (2016). Incorporation of mesoporous silica nanoparticles into random electrospun PLGA and PLGA/gelatin nanofibrous scaffolds enhances mechanical and cell proliferation properties. Mat. Sci. Eng. C-Mater.

[34] Sun DQ, Hussain HI, Yi ZF, Rookes JE, Kong LX, Cahill DM (2016). Mesoporous silica nanoparticles enhance seedling growth and photosynthesis in wheat and lupin. Chemosphere.

[35] McCarthy CA, Ahern RJ, Dontireddy R, Ryan KB, Crean AM (2016). Mesoporous silica formulation strategies for drug dissolution enhancement: A review. Expert Opin. Drug Deliv.

[36] Shi NQ, Lei YS, Song LM, Yao J, Zhang XB, Wang XL (2013). Impact of amorphous and semicrystalline polymers on the dissolution and crystallization inhibition of pioglitazone solid dispersions. Powder Technol.

[37] Kusakabe K, Ichiki K, Hayashi JI, Maeda H, Morooka S (1996). Preparation and characterization of silica—polyimide composite membranes coated on porous tubes for CO2 separation. J. Membrane Sci.

[38] Kioul A, Mascia L (1994). Compatibility of polyimide-silicate ceramers induced by alkoxysilane silane coupling agents. J. Non-Cryst. Solids.

